# High prevalence of resistance to first-line drugs and multidrug resistance in patients with tuberculosis and type 2 diabetes mellitus at a referral hospital in Peru

**DOI:** 10.1371/journal.pone.0337152

**Published:** 2026-08-20

**Authors:** Sergio Barboza-Panaifo, Italo Erazo-Cárdenas, Nallely V. Chapoñan-Agip, Anabell Tenorio-Quispe, Marlon Yovera-Aldana

**Affiliations:** 1 Universidad Científica del Sur, Lima, Perú; 2 Grupo de Investigación NEMECS: Neurociencias, Metabolismo, Efectividad Clínica y Sanitaria, Universidad Científica del Sur, Lima, Perú; The University of Georgia, UNITED STATES OF AMERICA

## Abstract

**Introduction:**

The coexistence of tuberculosis (TB) and type 2 diabetes mellitus (T2DM) poses an emerging public health challenge, especially in high-TB-burden settings such as Peru. This comorbidity has been associated to unfavorable clinical outcomes and a higher frequency of drug-resistant TB.

**Objective:**

To estimate the prevalence of resistance to first-line antituberculosis drugs and identify factors associated with multidrug resistance tuberculosis (MDR-TB) among patients with T2DM and pulmonary TB treated at a public hospital in Lima, Peru.

**Methods:**

A cross-sectional study was conducted using secondary data from 130 adults with T2DM and microbiologically confirmed pulmonary TB treated at a Peruvian hospital between 2015 and 2019. Drug susceptibility results were categorized as susceptible, monoresistant, polyresistant, or MDR-TB. Factors associated with MDR and resistance to at least one first-line drug were evaluated using Poisson regression models with robust variance.

**Results:**

The prevalence of resistance to at least one first-line drug was 34.6%, and the prevalence of MDR-TB was 21.5%. The most frequent resistances were to isoniazid (28.4%) and rifampicin (26.2%). In the multivariable analysis, age 40–49 years (aPR = 2.99; 95% CI: 1.00–8.97) and age 70–85 years (aPR = 4.13; 95% CI: 1.16–14.7) and were independently associated with MDR-TB. Furthermore, a diabetes duration of at least 5 years was associated with resistance to at least one drug (aPR ≈ 2.1).

**Conclusion:**

A high prevalence of drug resistance, including MDR-TB was observed among patients with T2DM and pulmonary TB. Older age and longer duration of diabetes were associated with bacterial resistance in the adjusted analysis. These findings underscore the importance of considering diabetes-related clinical characteristics when evaluating drug resistance patterns in patients with tuberculosis.

## Introduction

On a global scale, tuberculosis (TB) has shown a sustained decline in both incidence and overall mortality since 1990, largely attributable to the implementation of the Directly Observed Treatment, Short-Course (DOTS) strategy. [1,2] Nevertheless, this decrease has been less pronounced in developing regions [3–5]. In Latin America, TB incidence has also declined, although important challenges persist. In Brazil, the incidence decreased from 42.8 to 35.2 cases per 100,000 population between 2001 and 2017, but increased again to 39.8 cases in 2023, particularly among vulnerable populations, exceeding the World Health Organization (WHO) target of 6.7 per 100,000 population [6,7]. Peru accounts for 14% of all TB cases in South America, with Lima Metropolitan Area and Callao reporting 55.6% of national cases [8]. Despite reductions in new cases during previous decades [9], the persistently high burden in specific regions underscores the need for tailored public health strategies.

One contributing factor to this limited progress is the increasing resistance to first-line anti-tuberculosis drugs [10–12]. Resistance patterns vary according to the number of affected drugs and their therapeutic relevance. Multidrug-resistant tuberculosis (MDR-TB), defined as resistance to both isoniazid and rifampicin, has been associated with poorer clinical outcomes. In a region of central Peru, the prevalence of MDR-TB was estimated at 3.7%, with 46% of these cases having received previous treatment and 59% experiencing treatment failure [13]. In Lima, another study reported an MDR-TB prevalence of 13% [14], reflecting a substantial disease burden of resistand disease.

The coexistence of TB and diabetes mellitus (DM) represents an important public health concern. Chronic hyperglycemia and associated immunometabolic alterations may impair host immune responses against *Mycobacterium tuberculosis,* increase susceptibility to infection, delay microbiological clearance, and contribute to suboptimal therapeutic responses, potentially favoring the emergence of drug-resistant strains [15–17] Globally, the estimated incidence of TB among individuals with DM is 129 cases per 100,000 person-years and 511 cases per 100,000 population [18]. In Lima, 12% of MDR-TB cases occur among patients with DM, and this proportion rises to 28% among those with a history of previous TB treatment [19]. Because patients with DM frequently have prior TB episodes and repeated exposure to anti-tuberculosis treatment, they may represent a population at increased frequency of drug resistance. However, data describing resistance patterns in this population remain limited in Peru. Therefore, this study aimed to describe the frequency of resistance to first-line anti-tuberculosis drugs and explore factors associated with MDR-TB, distinguishing between resistance to at least one first-line drug and multidrug resistance, among adults with DM treated at a specialized public referral hospital in Lima, Peru.

## Materials and methods

### Study design and setting

We conducted a cross-sectional descriptive study based on a secondary analysis of a clinical database from the” Divino Niño” Center of Excellence for Tuberculosis at Maria Auxiliadora Hospital (HMA) in Lima, Peru. This high-capacity referral center is part of the Peruvian Ministry of Health and serves patients from 118 primary care facilities across southern Lima. The population covered is predominantly of low socioeconomic status and receives subsidized public health insurance. The Center of Excellence manages complex tuberculosis cases, including drug-resistant TB and TB associated with comorbidities such as diabetes mellitus. Data collection and analysis were carried out during the second half of 2021.

### Study population, sample, and sampling strategy

Patients aged 18 years or older with a diagnosis of type 2 diabetes mellitus (T2DM) were included. The diagnosis was confirmed based on medical history, self-report, use of hypoglycemic agents, or diagnosis at admission according to the American Diabetes Association (ADA) criteria. In addition, participants were required to have active pulmonary tuberculosis confirmed by drug susceptibility testing for anti-tuberculosis agents, performed within the first two months of treatment. Furthermore, only patients treated between 2015 and 2019 were considered. Patients with extrapulmonary tuberculosis, HIV infection, or inconsistent clinical records were excluded. We conducted a census of all eligible patients treated during the study period; no sampling was performed.

### Variables

#### Resistance classification.

Bacterial resistance was defined according to the number and therapeutic hierarchy of the four main anti-tuberculosis drugs, and was classified into four categories. Multidrug-resistant tuberculosis (MDR-TB) was defined as resistance to both isoniazid and rifampicin. Polyresistance was defined as resistance to more than two drugs, excluding the combination of isoniazid and rifampicin. Monoresistance was defined as resistance to any one of the four drugs in isolation, and drug-susceptible tuberculosis was defined as susceptibility to all four drugs. Data were extracted by endocrinology service physicians from reports provided by the National Institute of Health (INS) and documented in the patients’ medical records. The INS performs drug susceptibility testing (DST) in accordance with World Health Organization (WHO) criteria and the 2019 Peruvian Technical Standard. For the isolation of *Mycobacterium tuberculosis*, the absolute concentration method on Löwenstein–Jensen medium is used. The concentrations used for susceptibility testing were as follows: isoniazid, 0.2 μg/mL; rifampicin, 40 μg/mL; pyrazinamide, 100 μg/mL; and ethambutol, 2 μg/mL. For the purposes of this study, two primary outcomes were defined. The first was resistance to at least any first-line drug (monoresistance, multidrug resistance, or polyoresistance classified as “yes,” and drug-susceptible cases classified as “no”). The second was multidrug resistance (yes or no).

#### Other variables.

Sociodemographic variables were categorized as follows: age (23–39 years, 40–49 years, 50–59 years, 60–69 years, and 70–85 years); sex (male, female); educational level (primary/secondary, higher); and place of residence (San Juan de Miraflores, Villa María del Triunfo, Villa El Salvador, Chorrillos, and other districts). Clinical variables included: duration of diabetes diagnosis (≤5 years, ≥ 5 years); body mass index (BMI) (<25 kg/m^2^, 25–29.9 kg/m^2^, ≥ 30 kg/m^2^); history of tuberculosis (yes, no); diabetes treatment regimen (diet only, metformin only, basal insulin ± metformin, and basal-bolus insulin regimen); and adherence to hypoglycemic treatment, defined as compliance with the prescribed dosage (yes, no). Fasting plasma glucose levels (<130 mg/dL, 130–200 mg/dL, ≥ 200 mg/dL), postprandial glucose levels (<140 mg/dL, 140–180 mg/dL, ≥ 180 mg/dL), and glycemic control according to HbA1c (≤7%, 7–9%, ≥ 9%) from the most recent month on record were also described.

### Procedures and analysis plan

Approval was obtained from the Office for Research and Teaching Support of the HMA, as well as from the coordination of the Endocrinology Service. Access to the database was granted on August 1, 2021, and the analysis was conducted through December 31, 2021. Data were exported from Microsoft Excel into Stata statistical software, version 16.0 (StataCorp, College Station, TX, USA).

For the analysis, categorical variables were summarized using absolute and relative frequencies. Age and HbA1c were summarized using the mean and standard deviation, whereas duration of diabetes was presented as the median and interquartile range due to its non-normal distribution. Associations between demographic and clinical variables and the two primary outcomes, resistance to at least one first-line drug and multidrug resistance, were assessed using Pearson’s chi-square test or Fisher’s exact test, when appropriate.

In the multivariable analysis, generalized linear models with a log link and robust Poisson variance were used to estimate crude and adjusted prevalence ratios (PRs) with 95% confidence intervals. This approach was selected because the prevalence of the outcomes exceeded 10%, which could lead to overestimation of effect measures derived logistic regression models. Given the limited number of MDR-TB cases and the exploratory nature of the study, the adjusted models included a restricted number of variables selected according to clinical relevance and strength of association in the bivariable analysis, in order to reduce the risk of overfitting. Multicollinearity among covariates was evaluated in the adjusted models.

Sample adequacy was evaluated based on the precision of prevalence estimates for MDR-TB and resistance to at least one first-line drug resistance among patients with T2DM and pulmonary TB. Using EPIDAT version 4.2, the estimated precision for the prevalence of MDR-TB (21.5%) was ± 7.1%, and for resistance to at least one first-line drug (34.6%) was ± 8.2%, assuming a 95% confidence level and a final analytical sample of 130 subjects [20].

### Ethics statement

The research protocol was approved by the Ethics Committees of María Auxiliadora Hospital (HMA/CIEI/0016/2021) and Universidad Científica del Sur (065–2021-PRE15). Because this was a retrospective study based on secondary analysis of anonymized clinical data, the requirement for informed consent was waived by the ethics committees. The database did not contain personally indentifiable information.

## Results

### Subject selection

**Fig 1:**
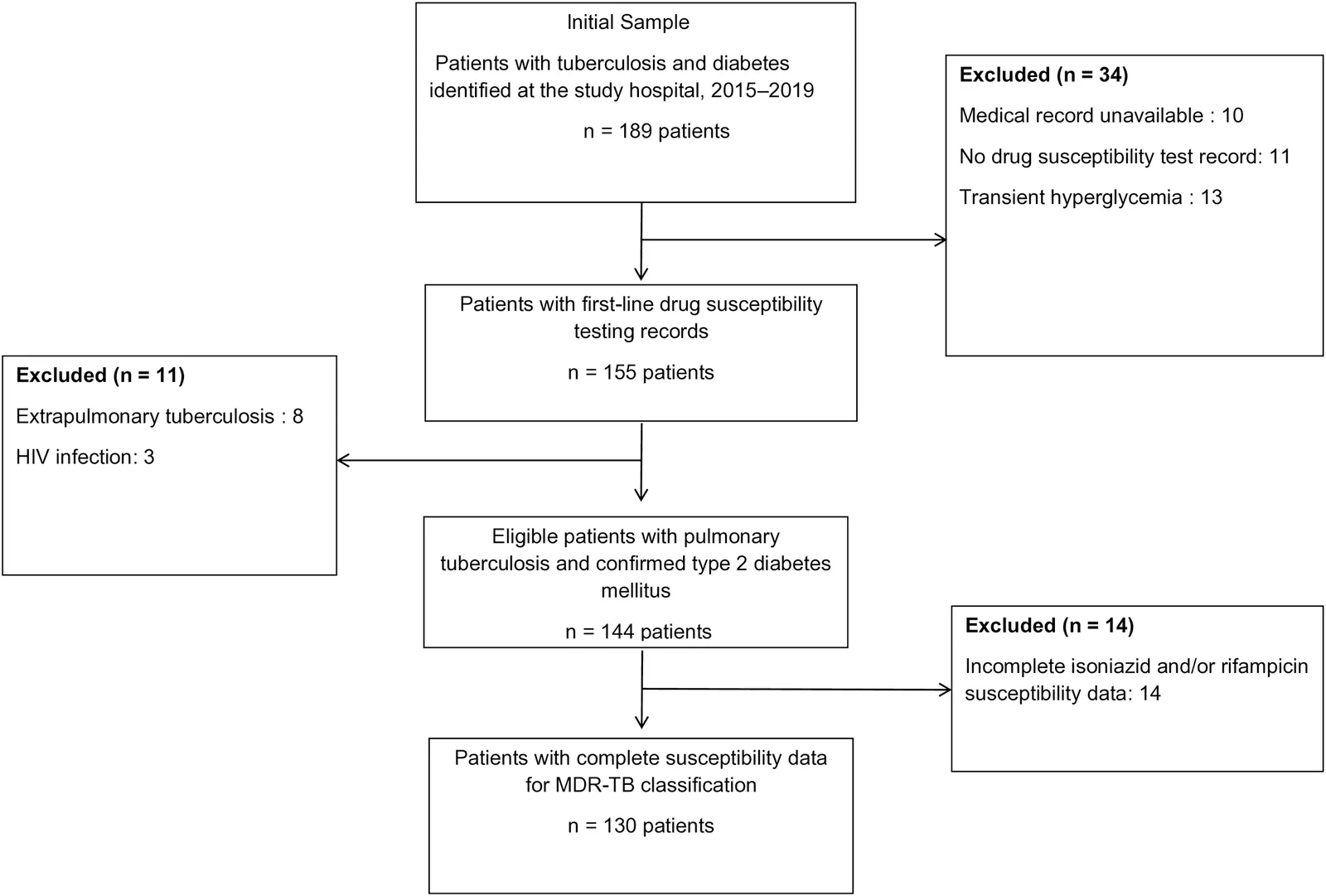
Flow diagram of patient selection for the study on multidrug-resistant tuberculosis (MDR-TB) and type 2 diabetes mellitus. Among the 130 patients with complete data, 28 (21.5%) had MDR-TB and 45 (34.6%) showed resistance to at least one first-line drug.

From 2015 and 2019, a total of 189 unique patients diagnosed with both diabetes and tuberculosis were treated. After applying the eligibility criteria in the database, a total of 130 patients with complete susceptibility data for MDR-TB classificationsubjects were included (Fig 1).

### General characteristics

Of the 130 patients included in the final analytical sample, 54.6% were male, and the largest age group was 50–59 years, accounting for 33.1% of participants. Most participants (54.6%) had completed primary or secondary education.

Regarding clinical characteristics, 36.7% of individuals were overweight and 13.9% were obese. In addition, 79.2% had a history of previous tuberculosis, and 41.7% had been diagnosed with diabetes for less than 5 years. Regarding diabetes treatment, 55.1% of participants used a basal insulin ± metformin regimen while 35.4% used a basal-bolus insulin regimen.

Poor glycemic control was common in the study population. Overall 86.6% had fasting blood glucose levels ≥130 mg/dL and 88.9% had postprandial blood glucose levels ≥180 mg/dL. Regarding glycated hemoglobin (HbA1c), 15.8% had values between 7% and 9%, while 71.1% had HbA1c levels ≥ 9% (**Table 1).**

**Table 1:** Sociodemographic and clinical characteristics of patients included in the final analytical sample for MDR-TB analysis.

Characteristics	Patients with complete data for MDR (N = 130)
**Demographics**	
**Sex (n = 130)**	
Male	71 (54.6)
Female	59 (45.4)
**Age group, years (n = 130)**	
23–39	25 (19.2)
40–49	31 (23.9)
50–59	43 (33.1)
60–69	23 (17.7)
70–85	8 (6.2)
Mean ± SD	50.6 ± 12.1
**Education level (n = 130)**	
Primary or secondary	71 (54.6)
Higher education	59 (45.4)
**District of residence (n = 130)**	
San Juan de Miraflores	40 (30.8)
Villa María del Triunfo	31 (23.9)
Villa el Salvador	25 (19.2)
Chorrillos	16 (12.3)
Other	18 (13.9)
**Clinical**	
**Body Mass Index (n = 130)**	
Underweight (<18.5 kg/m²)	17 (13.1)
Normal (18.5–24.9 kg/m²)	48 (36.9)
Overweight (25.0–29.9 kg/m²)	47 (36.2)
Obese (≥30 kg/m²)	18 (13.9)
**Prior tuberculosis (n = 130)**	
Present	103 (79.2)
Absent	27 (20.8)
**Duration of diabetes, years (n = 127)**	
<5 years	53 (41.7)
5-9.9 years	39 (30.7)
≥ 10 years	35 (27.6)
Median [IQR]	5 [2–10]
**Diabetes treatment regimen (n = 127)**	
Diet only	6 (4.7)
Metformin only	6 (4.7)
Basal insulin ± metformin	70 (55.1)
Basal-bolus insulin regimen	45 (35.4)
**Adherence to hypoglycemic treatment (n = 118)**	
Adherent	95 (80.5)
Non-adherent	23 (19.5)
**Fasting blood glucose (n = 127)**	
<130 mg/dL	17 (13.4)
130–200 mg/dL	40 (31.5)
≥ 200 mg/dL	70 (55.1)
**Postprandial blood glucose (n = 117)**	
<140 mg/dL	6 (5.1)
140–180 mg/dL	7 (6.0)
≥180 mg/dL	104 (88.9)
**HbA1c (n = 114)**	
<7%	15 (13.2)
7–9%	18 (15.8)
≥9%	81 (71.1)
Mean ± SD	10.7 ± 3.0

Data are presented as n (%), unless otherwise indicated. N represents the number of patients with available data for each variable. The analytical sample for MDR-TB classification included 130 patients with complete isoniazid and rifampicin susceptibility data. “Primary or secondary” includes participants with no formal education, preschool, primary, or secondary schooling.“Other districts” include Surco, Barranco, Lurín, Pachacámac, San Bartolo, Pucusana, Punta Negra, Punta Hermosa, and Santa María del Mar.

### Prevalence of bacterial resistance to first-line drugs

The first-line drug with the highest prevalence of resistance was isoniazid, affecting 28.4% of patients (95% CI = 21.9–37.0), followed by rifampicin with 26.2% (95% CI = 18.8–34.6). Overall, resistance to at least one first-line drug was observed in 34.6% of participants (95% CI = 26.5–43.4). The prevalence of MDR-TB, polyresistance, and monoresistance were 21.5% (95% CI = 14.8–29.6), 2.3% (95% CI = 0.4–6.5), and 10.8% (95% CI = 6.0–17.4), respectively (Table 2).

**Table 2:** Resistance to first-line antituberculosis drugs in patients with type 2 diabetes mellitus.

Resistance pattern	n	Prevalence (95% IC)
**Drug-specific resistance (non-mutually exclusive)**		
Isoniazid (H)	37	28.4 (21.9–37.0)
Rifampicin (R)	34	26.2 (18.8–34.6)
Pyrazinamide (Z)	17	13.1 (7.8–20.1)
Ethambutol (E)	17	13.1 (7.8–20.1)
**Resistance to at least one first-line drug**	45	34.6 (26.5–43.4)
**Multidrug-resistant TB (MDR-TB)** **(resistance to at least H + R)**	28	21.5 (14.8–29.6)
H + R	13	
H + R + E	2	
H + R + Z	2	
H + R + E + Z	11	
**Polyresistance**	3	2.3 (0.4–6.5)
H + E + Z	3	
**Monoresistance**	14	10.8 (6.0–17.4)
H only	6	
R only	6	
E only	1	
Z only	1	

Data are expressed as number of cases (n) and prevalence (%; 95% confidence interval). Drug-specific resistance categories are not mutually exclusive; a single patient may contribute to more than one category. MDR-TB was defined as concurrent resistance to isoniazid (H) and rifampicin (R), with or without resistance to other first-line drugs (E, Z). Polyresistance was defined as resistance to more than one first-line drugs, excluding concurrent resistance to isoniazid and rifampicin. Monoresistance was defined as resistance to a single first-line drug.

### Factors associated with TB-MDR

In the bivariate analysis, the 50–59 age group had the lowest prevalence of MDR-TB (9.3%). A J-shaped distribution was observed, with higher prevalence at the age extremes, particularly among individuals aged 70–85 years (50.0%; p = 0.039). No significant differences were observed for the remaining variables **(Table 3).**

**Table 3:** Sociodemographic and clinical characteristics associated with multidrug-resistant tuberculosis (MDR-TB) in patients with type 2 diabetes mellitus and pulmonary tuberculosis.

Characteristics	MDR-TB positive n (%)	MDR-TB negative n (%)	p-value	Resistance to at least one first-line drug n (%)	Fully Susceptible n (%)	p-value
**Sex (n = 130)**						
Male	15 (53.6)	56 (54.9)	0.900 ^a^	22 (48.9)	49 (57.6)	0.340 ^a^
Female	13 (46.4)	46 (45.1)		23 (51.1)	36 (42.4)	
**Age group, years (n = 130)**						
23 - 39	7 (25.0)	18 (17.6)	0.039 ^a^	10 (22.2)	15 (17.6)	0.219 ^a^
40 - 49	9 (32.1)	22 (21.6)		12 (26.7)	19 (22.4)	
50 - 59	4 (14.3)	39 (38.2)		9 (20.0)	34 (40.0)	
60 - 69	4 (14.3)	19 (18.6)		10 (22.2)	13 (15.3)	
70 - 85	4 (14.3)	4 (3.9)		4 (8.9)	4 (4.7)	
**Education level (n = 130)**						
Primary or secondary	13 (46.4)	58 (56.9)	0.326 ^a^	20 (44.4)	51 (60.0)	0.090 ^a^
Higher education	15 (53.6)	44 (43.1)		25 (55.6)	34 (40.0)	
**District of residence (n = 130)**						
San Juan de Miraflores	9 (32.1)	31 (30.4)	0.926 ^a^	13 (28.9)	27 (31.8)	0.845 ^a^
Villa María del Triunfo	8 (28.6)	23 (22.5)		12 (26.7)	19 (22.4)	
Villa El Salvador	4 (14.3)	21 (20.6)		7 (15.6)	18 (21.2)	
Chorrillos	3 (10.7)	13 (12.7)		7 (15.6)	9 (10.6)	
Other*	4 (14.3)	14 (13.7)		6 (13.3)	12 (14.1)	
**Body mass index (n = 130)**						
Underweight (< 18.5 kg/m²)	7 (25.0)	10 (9.8)	0.120 ^a^	7 (15.6)	10 (11.8)	0.550 ^a^
Normal (18.5–24.9 kg/m²)	7 (25.0)	41 (40.2)		17 (37.8)	31 (36.5)	
Overweight (25.0–29.9 kg/m²)	9 (32.1)	38 (37.3)		13 (28.9)	34 (40)	
Obese (≥ 30 kg/m²)	5 (17.9)	13 (12.7)		8 (17.8)	10 (11.8)	
**Duration of diabetes, years (n = 127)**						
<5	7 (26.9)	46 (45.5)	0.193 ^a^	11 (25.6)	42 (50.0)	0.031 ^a^
5 a 9.9	9 (34.6)	30 (29.7)		17 (39.5)	22 (26.2)	
≥ 10	10 (38.5)	25 (24.8)		15 (34.9)	20 (23.8)	
**Prior tuberculosis (n = 130)**						
Present	22 (78.6)	81 (79.4)	0.923 ^a^	36 (80.0)	67 (78.8)	0.875 ^a^
Absent	6 (21.4)	21 (20.6)		9(20)	18(21.2)	
**Diabetes treatment regimen (n = 127)**						
Diet only	1 (3.8)	5 (5.0)	0.907 ^b^	2 (4.7)	4 (4.8)	0.797 ^b^
Metformin only	1 (3.8)	5 (5.0)		1 (2.3)	5 (6.0)	
Basal insulin ± metformin	16 (61.5)	54 (53.5)		26 (60.5)	44 (52.4)	
Basal-bolus insulin regimen	8 (30.8)	37 (36.6)		14 (32.6)	31 (36.9)	
**Adherence to hypoglycemic treatment (n = 118)**						
Adherent	16 (69.6)	79 (83.2)	0.140 ^a^	29 (74.4)	66 (83.5)	0.236 ^a^
Non-adherent	7 (30.4)	16 (16.8)		10 (25.6)	13 (16.5)	
**Fasting blood glucose,** (n = 127)						
< 130 mg/dL	1 (3.8)	16 (15.8)	0.277 ^a^	4 (9.3)	13 (15.5)	0.591 ^a^
130 - 200 mg/dL	9 (34.6)	31 (30.7)		15 (34.9)	25 (29.8)	
≥ 200 mg/dL	16 (61.5)	54 (53.5)		24 (55.8)	46 (54.8)	
**HbA1c, (n = 114)**						
< 7%	3 (12.5)	12 (13.3)	0.988 ^a^	6 (15.8)	9 (11.8)	0.407 ^a^
7-9%	4 (16.7)	14 (15.6)		8 (21.1)	10 (13.2)	
≥ 9%	17 (70.8)	64 (71.1)		24 (63.2)	57 (75.0)	

Data are expressed as n (%) unless otherwise indicated. Percentages are presented by column. p-values were calculated using Pearson’s chi-square test (ᵃ) or Fisher’s exact test (ᵇ), as appropriate. N represents the number of participants with available data for each variable. “Other districts” include Surco, Barranco, Lurín, Pachacámac, San Bartolo, Pucusana, Punta Negra, Punta Hermosa, and Santa María del Mar. MDR-TB: multidrug-resistant, defined as resistance to at least isoniazid and rifampicin. Resistance to at least one first-line drug included MDR-TB, polyresistance, and monoresistance.

In the multivariate analysis, compared with the 50–59 years age group, which had the lowest prevalence and was used as the reference, individuals aged 40–49 and 70–85 years showed higher adjusted prevalence ratios for MDR-TB. Specifically, the prevalence of MDR-TB was approximately threefold higher among those aged 40–49 years (aPR = 2.99; 95% CI = 1.00–8.97; p = 0.049) and more fourfold higher among those aged 70–85 years (aPR = 4.13; 95% CI = 1.16–14.7; p = 0.028). These estimates were adjusted for age group and body mass index (**Table 4).**

**Table 4:** Multivariable analysis of factors associated with multidrug-resistant tuberculosis (MDR-TB) and resistance to at least one first-line drug in patients with type 2 diabetes mellitus and pulmonary tuberculosis.

	MDR-TB				Resistance to at least one first-line drug			
Factors (n = 130)	cPR (CI 95%)	p-value	aPR^a^ (CI 95%)	p-value	cPR (CI 95%)	p-value	aPR^b^ (CI 95%)	P-value
**Sex, (n = 130)**								
Male	Ref.				Ref.			
Female	1.04 (0.53–2.01)	0.901			1.26 (0.78–2.02)	0.342		
**Age group, years (n = 130)**								
23–39	3.01(0.97–9.31)	0.056	2.62 (0.85–8.05)	0.091	1.91 (0.89–4.07)	0.093	1.69 (0.75–3.81)	0.205
40–49	3.12 (1.05–9.26)	0.040	2.99 (1.00–8.97)	0.049	1.85 (0.89–3.85)	0.100	1.79 (0.88–3.64)	0.103
50–59	Ref.		Ref.		Ref.			
60–69	1.86 (0.51–6.82)	0.344	1.75 (0.51–6.08)	0.373	2.08 (0.98–4.39)	0.055	1.86 (0.92–3.80)	0.086
70–85	5.37 (1.67–17.30)	0.005	4.13 (1.16–14.70)	0.028	2.39 (0.96–5.92)	0.060	3.03 (1.14–8.05)	0.026
**Education level, (n = 130)**								
Primary or secondary*	Ref.				Ref.		Ref.	
Higher education	1.38 (0.71–2.69)	0.330			1.50 (0.93–2.43)	0.094	1.43 (0.88–2.32)	0.145
**District of residence, (n = 130)**								
San Juan de Miraflores	Ref.				1.19 (0.63–2.23)	0.587		
Villa María del Triunfo	1.14 (0.49–2.63)	0.747			0.86 (0.40–1.87)	0.706		
Villa El Salvador	0.71 (0.24–2.07)	0.533			1.35 (0.66–2.75)	0.416		
Chorrillos	0.83 (0.25–2.69)	0.761			1.02 (0.46–2.27)	0.950		
Other districts*	0.98 (0.34–2.80)	0.981			1.00			
**Body mass index, (n = 130)**								
Underweight (< 18.5 kg/m²)	2.82 (1.15–6.89)	0.023	2.19 (0.88–5.43)	0.089	1.16 (0.58–2.31)	0.667		
Normal (18.5–24.9 kg/m²)	Ref.				Ref.			
Overweight (25.0–29.9 kg/m²)	1.31 (0.53–3.24)	0.556	1.31 (0.54–3.17)	0.542	0.78 (0.42–1.43)	0.421		
Obese (≥ 30 kg/m²)	1.90 (0.69–5.25)	0.214	1.73 (0.62–4.78)	0.290	1.25 (0.66–2.39)	0.490		
**Duration of diabetes, years (n = 127)**								
< 5	Ref.				Ref.			
5–9.9	1.74 (0.71–4.29)	0.225			2.10 (1.10–3.98)	0.023	2.14 (1.15–4.01)	0.016
≥ 10	2.16 (0.90–5.16)	0.082			2.06 (1.07–3.97)	0.030	2.15 (1.14–4.07)	0.017
**Prior tuberculosis (n = 130)**								
Absent	Ref.				Ref.			
Present	0.96 (0.43–2.13)	0.923			1.05 (0.58–1.91)	0.876		
**Diabetes treatment regimen, (n = 127)**								
Diet only	0.73 (0.11–4.62)	0.738			0.90 (0.28–2.91)	0.857		
Metformin	0.72 (0.11–4.62)	0.738			0.45 (0.07–2.78)	0.389		
Basal insulin ± metformin	Ref.				Ref.			
Basal–bolus insulin regimen	0.77 (0.36–1.67)	0.519			0.84 (0.49–1.43)	0.515		
**Adherence to hypoglycemic treatment (n = 118)**								
Adherent	Ref.				Ref.			
Non–adherent	0.55 (0.25–1.19)	0.130			0.70 (0.40–1.22)	0.214		
**Fasting blood glucose, (n = 127)**								
<130 mg/dL	Ref.				Ref.			
130–200 mg/dL	3.82 (0.52–28.10)	0.187			1.59 (0.62–4.11)	0.336		
≥ 200 mg/dL	3.88 (0.54–27.50)	0.174			1.46 (0.58–3.66)	0.422		
**HbA1c, (n = 114)**								
< 7%	Ref.				Ref.			
7–9%	1.11 (0.29–4.22)	0.877			1.11 (0.49–2.50)	0.799		
≥ 9%	1.04 (0.34–3.15)	0.932			0.74 (0.36–1.50)	0.406		

cPR: crude prevalence ratio; aPR: adjusted prevalence ratio; CI: confidence interval. Adjusted prevalence ratios were estimated using Poisson regression models with robust variance. Due to the limited number of MDR-TB cases and the exploratory nature of the study, only variables with stronger associations in the bivariate analysis and clinical relevance were included in the adjusted models to reduce the risk of overfitting. Model a was adjusted for age group and body mass index. Model b was adjusted for age group, education level, and duration of diabetes. MDR-TB: multidrug-resistant tuberculosis, defined as resistance to at least isoniazid and rifampicin. Resistance to at least one first-line drug included mono-, poly-, and multidrug resistance to first-line agents. Reference categories are indicated as Ref.

### Factors associated with bacterial resistance to first-line drugs

In the bivariate analysis, participants with a duration of 5 years or longer showed a high prevalence of resistance to at least first-line drug (p = 0.031). No significant differences were observed for the remaining variables (**Table 3).**

In the multivariate analysis, compared with participants with a diabetes duration of less than 5 years, those with diabetes durations of 5–9.9 years and 10 years or longer showed approximately a twofold high prevalence of resistance to at least first-line drug (aPR = 2.14; 95% CI = 1.15–4.01; p = 0.016) and (aPR = 2.15; 95% CI = 1.14–4.07; p = 0.017), respectively. In addition, individuals aged 70–85 years had a threefold higher prevalence of resistance to at least one first-line drug compared with those aged 50–59 years (aPR 3.03; 95% CI = 1.14–8.05; p = 0.026). These estimates were adjusted for age group, duration of diabetes, and education level (**Table 4).**

## Discussion

### Principal findings

This study documents a high frequency of bacterial resistance to first-line anti-tuberculosis drugs among patients with tuberculosis and type 2 diabetes mellitus (TB-DM) co-infection. A total of 34.6% of the cases exhibited resistance to at least one pharmaceutical agent, while 21.5% had MDR-TB. These proportions were higher than those reported for the general tuberculosis population in Peru. Older age and longer duration of diabetes were associated with bacterial resistance in the adjusted analyses.. By contrast, there was no significant association between resistance and variables such as sex, glycemic control, history of previous tuberculosis, body mass index (BMI) and adherence to hypoglycemic treatment.

### Comparison with other studies

Various studies have documented disparate rates of anti-tuberculosis drug resistance among patients with TB-DM. In a Mexican cohort of patients with type 2 diabetes, 14% had MDR and 19% had resistance to at least one first-line drug [21]. The prevalence of MDR in Indonesia was 17% [22]. Similarly, a study in China reported an MDR-TB prevalence of 6.6% and resistance to at least one drug in 23% of cases [23]. In Perú, a previous study among patients with TB-DM, reported 15.6% MDR-TB in 15.6% of cases and resistance to at least one drug in 9.1% [19]. Differences in study populations, healthcare settings, and methodologies may explain the variation in resistance frequencies across studies.

Previous studies have reported that longer duration of diabetes, previous tuberculosis, and poor glycemic control are associated with MDR-TB [24,25]. In our study, duration of diabetes was significantly associated with resistance to at least one first-line drug. However, no significant associations were found with previous tuberculosis or HbA1c levels. One possible explanation is the limited variability of these variables in our sample, given the high proportion of patients with previous tuberculosis (>80%) and poor glycemic control (87% with HbA1c ≥ 7%). In addition, because this study was conducted at a specialized referral center for complex and drug-resistant tuberculosis cases, the prevalence estimates reported here should not be interpreted as representative of the overall TB-DM population in Peru. This setting may have concentrated patients with more severe disease, previous treatment exposure, and higher baseline probabilities of drug resistance.

Previous studies have also suggested a higher frequency of resistance among middle-aged adults, particularly those aged 45–64 years, possibly related to greater community exposure and lower treatment adherence [24]. In contrast, our study identified a higher prevalence of resistance among adults 70 years or older. This finding may reflect the combined effect of comorbidities, immunological changes associated with aging, and previous exposure to tuberculosis treatment.

### Immunological and pharmacological mechanisms

The high prevalence of MDR-TB observed in this study may be related to multiple clinical and biological factors. First, most patients had a history of previous tuberculosis treatment, suggesting a high proportion of retreatment cases, which has beeb associated with an increased probability of resistant strains [24]. From an immunological perspective, T2DM induces a state of chronic immune dysfunction associated with sustained hyperglycemia. This phenomenon affects multiple components of the immune response, including neutophils, macrophages, and T lymphocytes, as well as the production of reactive oxygen species (ROS) [26]. Furthermore, non-enzymatic glycation of proteins, including immunoglobins may impair the immune response against *Mycobacterium tuberculosis*, potentially contributing to higher bacterial burden, greater radiological involvement, and delayed bacteriological conversion [27]. Pharmacokinetic studies suggest that patients with DM may have reduced plasma concentrations of rifampicin and isoniazid. Proposed mechanisms include gastroparesis, hepatic metabolic alterations and increased renal clearance [28]. Moreover, drug interactions, such as those observed described between rifampicin and metformin, may impair glycemic control and contribute to persistent hyperglycemia, and reduced therapeutic response [29].

The association between older age and resistance may reflect age-related immune changes (immunosenescence), a greater burden of comorbidities, and previous exposure to anti-tuberculosis treatment [24]. Similarly, longer duration of diabetes has been associated with cumulative immune dysfunction, and alterations in drug absorption or metabolism [30]. Although no statistically significant association was observed between HbA1c and resistance, 71% of patients with TB-DM had HbA1c levels ≥9%, suggesting that poor glycemic control could still play a role in bacterial resistance. Previous studies have reported significant associations between HbA1c ≥ 9% and MDR-TB [24,25]. A similar non-significant pattern was observed for BMI, where undernutrition and obesity showed higher frequencies of resistance compared with normal BMI categories. Undernutrition may impair immune function, whereas obesity may contribute through chronic inflammation and insulin resistance.

### Adherence to hypoglycemic treatment

Treatment regimens for TB-DM are frequently complex, which may contribute to reduced treatment adherence, particularly in the presence of socioeconomic barriers and disease-related stigma [31]. In addition, patients with diabetes may experience adverse drug reactions more frequently, potentially affecting treatment continuation [32]. Poor adherence to treatment among patients with TB-DM has been associated with higher mortality and increased complications [33]. Insulin remains an important therapeutic option in TB-DM because of its safety profile and glycemic efficacy; however, limited access in low-resource settings may negatively affect adherence. For this reason, pharmacological treatment should ideally be accompanied by interventions focused on diet and physical activity to improve glycemic control and support tuberculosis treatment outcomes [34]. Integrated care models for tuberculosis and diabetes may improve the management of both conditions, although their implementation remains challenging in centralized health systems [35].

### Implications for public health

The increasing burden of TB-DM comorbidity represents an important public health challenge, particularly in low- and middle-income countries. DM has been associated with worse clinical outcomes, including treatment failure, relapse and drug resistance [15,36]. Although our study did not evaluate public health interventions, the high frequency of bacterial resistance among patients with TB-DM highlights the need for approaches to tuberculosis and diabetes care. Potential strategies may include bidirectional screening, and closer clinical monitoring of patients with TB-DM, particularly in socially vulnerable populations, where the coexistence of both conditions may increase healthcare utilization and economic burden [37].

### Strengths and limitations

This study has several limitations. Its retrospective cross-sectional design is primarily descriptive and aimed at estimating prevalences; therefore, the associations identified should be interpreted cautiously and cannot establish causality. Additionally, missing data for variables such as HbA1c, treatment adherence, and glycemic measures limited some analyses. Moreover, plasma concentrations of anti-tuberculosis drugs and pharmacokinetic or pharmacodynamic parameters were not available, precluding a more detailed evaluation of mechanisms potentially associated with drug resistance. The relatively small number of MDR-TB cases restricted the number of variables that could be included in the adjusted models, increasing the possibility of residual confounding. Finally, because the study was conducted at a specialized referral center for drug-resistant tuberculosis and comorbidities, the findings may not be generalizable to other healthcare settings.

Nevertheless, the study also has important strengths. The inclusion of patients from a specialized referral center provided access to clinically complex cases with a high burden of drug resistance and comorbidities. Laboratory diagnoses were based on drug susceptibility testing validated by the Peruvian National Institute of Health, supporting the reliability of the microbiological data. In addition, the use of adjusted multivariable models facilitated the assessment of factors associated ith bacterial resistance while accounting for potential confounders. The inclusion of all eligible patients through a census approach minimized sampling error and enhanced the internal validity of the study findings.

### Research recommendations

Prospective studies are needed to evaluate whether intensive glycemic control improves tuberculosis treatment outcomes. Pharmacokinetic evaluations could help determine if dose adjustments or individualized rifampicin and isoniazid regimens are warranted in patients with diabetes. Further research should also explore immune responses in TB-DM comorbidity, including macrophage function, T-cell activity and cytokine profiles, as well as the potential rol of genomic tools in distinguishing acquired from transmitted MDR-TB. In addition, future studies should assess the effectiveness and feasibility of integrated public health strategies, such as combined TB-DM clinics and patient-centered programs, particularly in resource-limited settings. These interventions may help strengthen therapeutic adherence and support self-care.

## Conclusions

A high prevalence of resistance to first-line anti-tuberculosis drugs, including MDR-TB, was observed among patients with TB-DM. Older age and longer duration of diabetes were associated with bacterial resistance in the adjusted analysis. These findings highlight the burden of drug resistance among patients with TB-DM treated at a specialized referral center in Peru.

## Supporting information

S1 Table Comparison between the initial database and the final analytical sample included for MDR-TB classification. (DOCX)

S1 File Analysis dataset. (XLSX)
