# Supplementary material for: High prevalence of resistance to first-line drugs and multidrug resistance in patients with tuberculosis and type 2 diabetes mellitus at a referral hospital in Peru
Source: PLoS One. 2026 Aug 20;21(8):e0337152. doi: 10.1371/journal.pone.0337152 (PMC13492757; doi:10.1371/journal.pone.0337152)
Supplement: Supplementary file 1 [file pone.0337152.s001.docx]

**S1 Table. Comparison Between the Initial Database and the Final Analytical Sample Included for MDR-TB Classification**

| Characteristics | Initial database (N=144) | Patients with complete susceptibility data for MDR-TB classification (N=130) |
| --- | --- | --- |
| **Demographics** |  |  |
| **Sex** |  |  |
| Male | 78/144 (54.1) | 71/130 (54.6) |
| Female | 66/144 (45.9) | 59/130 (45.4) |
| **Age group, years** |  |  |
| 23–39 | 25/144 (17.1) | 25/130 (19.2) |
| 40–49 | 34/144 (23.3) | 31/130 (23.9) |
| 50–59 | 52/144 (35.6) | 43/130 (33.1) |
| 60–69 | 26/144 (17.8) | 23/130 (17.7) |
| 70–85 | 9/144 (6.2) | 8/130 (6.2) |
| Mean ± SD | 51.2 ± 11.8 | 50.6 ± 12.1 |
| **Education level** |  |  |
| Primary or secondary | 86/144 (59.6) | 71/130 (54.6) |
| Higher education | 58/144 (40.4) | 59/130 (45.4) |
| **District of residence** |  |  |
| San Juan de Miraflores | 43/144 (30.1) | 40/130 (30.8) |
| Villa María del Triunfo | 38/144 (26.7) | 31/130 (23.9) |
| Villa El Salvador | 27/144 (18.8) | 25/130 (19.2) |
| Chorrillos | 17/144 (11.8) | 16/130 (12.3) |
| Other districts | 19/144 (13.2) | 18/130 (13.9) |
| **Clinical characteristics** |  |  |
| **Body mass index** |  |  |
| Underweight (<18.5 kg/m²) | 19/144 (13.2) | 17/130 (13.1) |
| Normal (18.5–24.9 kg/m²) | 57/144 (39.6) | 48/130 (36.9) |
| Overweight (25.0–29.9 kg/m²) | 49/144 (34.0) | 47/130 (36.2) |
| Obesity (≥30 kg/m²) | 19/144 (13.2) | 18/130 (13.9) |
| **Prior tuberculosis** |  |  |
| Present | 117/144 (81.3) | 103/130 (79.2) |
| Absent | 27/144 (18.7) | 27/130 (20.8) |
| **Duration of diabetes, years** |  |  |
| <5 years | 62/143 (43.4) | 53/127 (41.7) |
| 5–9.9 years | 44/143 (30.8) | 39/127 (30.7) |
| ≥10 years | 37/143 (25.9) | 35/127 (27.6) |
| Median [IQR] | 5 [2–10] | 5 [2–10] |
| **Diabetes treatment regimen** |  |  |
| Diet only | 7/143 (4.9) | 6/127 (4.7) |
| Metformin only | 6/143 (4.2) | 6/127 (4.7) |
| Basal insulin ± metformin | 74/143 (51.8) | 70/127 (55.1) |
| Basal-bolus insulin | 56/143 (39.1) | 45/127 (35.4) |
| **Adherence to hypoglycemic treatment** |  |  |
| Adherent | 96/120 (80.0) | 95/118 (80.5) |
| Non-adherent | 24/120 (20.0) | 23/118 (19.5) |
| **Fasting blood glucose** |  |  |
| <130 mg/dL | 18/141 (12.7) | 17/127 (13.4) |
| 130–200 mg/dL | 47/141 (33.3) | 40/127 (31.5) |
| ≥200 mg/dL | 76/141 (54.0) | 70/127 (55.1) |
| **Postprandial blood glucose** |  |  |
| <140 mg/dL | 6/120 (5.0) | 6/117 (5.1) |
| 140–180 mg/dL | 7/120 (6.0) | 7/117 (6.0) |
| ≥180 mg/dL | 107/120 (89.0) | 104/117 (88.9) |
| **HbA1c** |  |  |
| <7% | 17/128 (13.3) | 15/114 (13.2) |
| 7–9% | 20/128 (15.6) | 18/114 (15.8) |
| ≥9% | 91/128 (71.1) | 81/114 (71.1) |
| Mean ± SD | 10.7 ± 3.0 | 10.7 ± 3.0 |

Abbreviations: HbA1c, glycated hemoglobin; IQR, interquartile range; MDR-TB, multidrug-resistant tuberculosis; SD, standard deviation.

Data are presented as n/N (%) unless otherwise indicated. N represents the number of patients with available data for each variable. The initial database included 144 patients, of whom 130 had complete susceptibility data required for MDR-TB classification. Primary or secondary education included participants with no formal education, primary school, or secondary school education. Other districts included Surco, Barranco, Lurín, Pachacámac, San Bartolo, Pucusana, Punta Negra, Punta Hermosa, and Santa María del Mar.
