# Supplementary material for: High prevalence of resistance to first-line drugs and multidrug resistance in patients with tuberculosis and type 2 diabetes mellitus at a referral hospital in Peru
Source: PLoS One. 2026 Aug 20;21(8):e0337152. doi: 10.1371/journal.pone.0337152 (PMC13492757; doi:10.1371/journal.pone.0337152)
Supplement: Peer review history

# Peer review: Decision Letter 0

**DOI:** 10.1371/journal.pone.0337152.r001

**Authors:** Christophe Sola

20 Apr 2026

Dear Dr. Yovera-Aldana,

Thank you for submitting your manuscript to PLOS ONE. After careful consideration, we feel that it has merit but does not fully meet PLOS ONE’s publication criteria as it currently stands. Therefore, we invite you to submit a revised version of the manuscript that addresses the points raised during the review process.

Please submit your revised manuscript by Jun 04 2026 11:59PM. If you will need more time than this to complete your revisions, please reply to this message or contact the journal office at plosone@plos.org. When you're ready to submit your revision, log on to https://www.editorialmanager.com/pone/ and select the 'Submissions Needing Revision' folder to locate your manuscript file.

- A letter that responds to each point raised by the academic editor and reviewer(s). You should upload this letter as a separate file labeled 'Response to Reviewers'.
- A marked-up copy of your manuscript that highlights changes made to the original version. You should upload this as a separate file labeled 'Revised Manuscript with Track Changes'.
- An unmarked version of your revised paper without tracked changes. You should upload this as a separate file labeled 'Manuscript'.

If you would like to make changes to your financial disclosure, please include your updated statement in your cover letter. Guidelines for resubmitting your figure files are available below the reviewer comments at the end of this letter.

If applicable, we recommend that you deposit your laboratory protocols in protocols.io to enhance the reproducibility of your results. Protocols.io assigns your protocol its own identifier (DOI) so that it can be cited independently in the future. For instructions see: https://journals.plos.org/plosone/s/submission-guidelines#loc-laboratory-protocols. Additionally, PLOS ONE offers an option for publishing peer-reviewed Lab Protocol articles, which describe protocols hosted on protocols.io. Read more information on sharing protocols at https://plos.org/protocols?utm_medium=editorial-email&utm_source=authorletters&utm_campaign=protocols.

As the corresponding author, your ORCID iD is verified in the submission system and will appear in the published article. PLOS supports the use of ORCID, and we encourage all coauthors to register for an ORCID iD and use it as well. Please encourage your coauthors to verify their ORCID iD within the submission system before final acceptance, as unverified ORCID iDs will not appear in the published article. *Only*  the individual author can complete the verification step; PLOS staff *cannot*  verify ORCID iDs on behalf of authors.

We look forward to receiving your revised manuscript.

Kind regards,

Christophe Sola, Pharm.D. Ph.D.

Academic Editor

PLOS One

Journal Requirements:

When submitting your revision, we need you to address these additional requirements.

1. Please ensure that your manuscript meets PLOS ONE's style requirements, including those for file naming. The PLOS ONE style templates can be found at

https://journals.plos.org/plosone/s/file?id=wjVg/PLOSOne_formatting_sample_main_body.pdf and

https://journals.plos.org/plosone/s/file?id=ba62/PLOSOne_formatting_sample_title_authors_affiliations.pdf

2. Please provide additional details regarding participant consent. In the ethics statement in the Methods and online submission information, please ensure that you have specified (1) whether consent was informed and (2) what type you obtained (for instance, written or verbal, and if verbal, how it was documented and witnessed). If your study included minors, state whether you obtained consent from parents or guardians. If the need for consent was waived by the ethics committee, please include this information.

If you are reporting a retrospective study of medical records or archived samples, please ensure that you have discussed whether all data were fully anonymized before you accessed them and/or whether the IRB or ethics committee waived the requirement for informed consent. If patients provided informed written consent to have data from their medical records used in research, please include this information

Once you have amended this/these statement(s) in the Methods section of the manuscript, please add the same text to the “Ethics Statement” field of the submission form (via “Edit Submission”).

For additional information about PLOS ONE ethical requirements for human subjects research, please refer to http://journals.plos.org/plosone/s/submission-guidelines#loc-human-subjects-research.

3. We note that your Data Availability Statement is currently as follows: [All relevant data are within the manuscript and its Supporting Information files.]

Please confirm at this time whether or not your submission contains all raw data required to replicate the results of your study. Authors must share the “minimal data set” for their submission. PLOS defines the minimal data set to consist of the data required to replicate all study findings reported in the article, as well as related metadata and methods (https://journals.plos.org/plosone/s/data-availability#loc-minimal-data-set-definition).

For example, authors should submit the following data:

- The values behind the means, standard deviations and other measures reported;

- The values used to build graphs;

- The points extracted from images for analysis.

Authors do not need to submit their entire data set if only a portion of the data was used in the reported study.

If your submission does not contain these data, please either upload them as Supporting Information files or deposit them to a stable, public repository and provide us with the relevant URLs, DOIs, or accession numbers. For a list of recommended repositories, please see https://journals.plos.org/plosone/s/recommended-repositories.

If there are ethical or legal restrictions on sharing a de-identified data set, please explain them in detail (e.g., data contain potentially sensitive information, data are owned by a third-party organization, etc.) and who has imposed them (e.g., an ethics committee). Please also provide contact information for a data access committee, ethics committee, or other institutional body to which data requests may be sent. If data are owned by a third party, please indicate how others may request data access.

4. If the reviewer comments include a recommendation to cite specific previously published works, please review and evaluate these publications to determine whether they are relevant and should be cited. There is no requirement to cite these works unless the editor has indicated otherwise.

[Note: HTML markup is below. Please do not edit.]

Reviewers' comments:

Reviewer's Responses to Questions

**Comments to the Author**

1. Is the manuscript technically sound, and do the data support the conclusions?

Reviewer #1: No

Reviewer #2: Yes

**********

2. Has the statistical analysis been performed appropriately and rigorously?

Reviewer #1: Yes

Reviewer #2: No

**********

3. Have the authors made all data underlying the findings in their manuscript fully available?

The PLOS Data policy

Reviewer #1: Yes

Reviewer #2: No

**********

4. Is the manuscript presented in an intelligible fashion and written in standard English?

Reviewer #1: No

Reviewer #2: No

**********

Reviewer #1: This manuscript addresses a public health issue, namely the burden of drug resistance among patients with pulmonary tuberculosis and type 2 diabetes mellitus in Peru. However, while the study provides useful descriptive data, several conceptual, methodological, and interpretative weaknesses limit the strength of the conclusions and require careful consideration.

Introduction:

The introduction adequately places the study within the broader global and regional context of tuberculosis

Methods:

The study population is drawn from a single, highly specialized referral centre that manages complex TB cases, including drug-resistant TB and patients with comorbidities. While this is clearly stated, the implications of this selection are insufficiently addressed throughout the manuscript. For example, the very high prevalence of MDR-TB and prior TB treatment (>80%) strongly suggests that the study population is not representative of the broader TB-DM population.

Regarding the four main anti-TB drugs, the fourth is pyrazinamide, not streptomycin, please clarify.

Diabetes treatment regimen classification is different between Methods section and Results section, please clarify.

Results:

Line 360: The authors mention “360 patients diagnosed with both diabetes and tuberculosis” during the study period. However, this number of patients is not mentioned anywhere else in the manuscript, not even in Figure 1. Please clarify.

Since the data in the "Total sample (N=144)" column of Table 1 are not mentioned in the text nor used anywhere in the manuscript, please delete this column or clarify how this data is relevant.

In Table 2, authors used HRZE abbreviations for anti-TB drugs but did not define all of them in the Table footnote. Furthermore, the abbreviation Z refers to pyrazinamide, but according to the Methods section, susceptibility to this antibiotic was not evaluated. Please clarify.

The authors analyse two outcomes: “Any drug resistance” and “MDR-TB”. However, since the MDR-TB category is included within the category any drug resistance, these two outcomes should not be treated and discussed separately in the main text.

In Table 3, percentages appear to be calculated by row rather than by column. While this presentation is mathematically correct, it makes it difficult to visually assess differences in the distributions between drug resistant positive and negative groups. A column-wise presentation (showing for example the distribution of age groups within each MDR-TB category) would be more intuitive and better aligned with the interpretation of Pearson’s chi-square test.

Lines 218-224: The authors say the same thing twice, just in different ways. Moreover, the youngest age group is not 40-49 years. Please rephrase. Please clarify the choice of variables for multivariable analysis.

Discussion:

The limitation related to recruitment from a specialized referral centre should be considered throughout the entire Discussion, rather than as an isolated point. This setting inherently selects for complex and drug-resistant TB cases, introducing substantial referral bias that affects prevalence estimates, observed associations, and their interpretation. The authors must revisit the entire discussion, systematically taking into account that this bias risk leading to an overinterpretation of the results and an extrapolation of the conclusions beyond the studied population. Moreover, in several places, the Discussion moves toward mechanistic explanations (immunosenescence, cumulative exposure, metabolic alterations) without sufficiently emphasizing the exploratory and observational nature of the findings.

Lines 255-256: The sentence “This study documents a high frequency of bacterial resistance to first-line anti-tuberculosis drugs among patients with tuberculosis and type 2 diabetes mellitus (TB-DM) co-infection” implies a comparative interpretation that is not directly supported by the study design. In the absence of a TB-only control group, the study cannot determine whether resistance is higher in TB–DM patients than in TB patients without diabetes. Moreover, recruitment from a specialized referral centre managing complex and drug-resistant TB cases introduces substantial selection bias, limiting the generalizability of the reported resistance frequency. Please rephrase.

Lines 270-271: Author state that: “However, the populations, levels of care, and methodologies included in those studies could explain the differences with our research.” Please elaborate further on this key point for the analysis of the study in light of the literature.

Lines 278-279: The manuscript refers to individuals aged 45-64 years as “young adults.” This terminology is misleading, as this age range is generally classified as middle-aged rather than young adulthood in epidemiological and clinical literature. Please use more appropriate age descriptors.

Line 346: The wording “clarified resistance patterns” is misleading. Multivariable analysis in this study does not resolve underlying resistance patterns but rather identifies exploratory associations within a selected referral population. Please rephrase.

Lines 358-361: Please specify more clearly what elements this study provides specifically.

General comments:

There are several typographical and grammatical errors throughout the manuscript that disrupt readability (spelling errors, inconsistent punctuation, use of the full word instead of the abbreviation).

There are sometimes discrepancies between the values in the main text and the Tables. Please correct them.

Authors used different terms to qualify resistance to at least one first-line anti-TB drug (any drug resistance, resistance to any first-line drug, any first-line drug resistance; of resistance to some first-line drug, resistance to at least one pharmaceutical agent, …) or resistance to at least two anti-TB drugs excluding MDR (oligoresistance and polyresistance), which is confusing. Please choose a single wording and keep it consistent throughout the manuscript.

This manuscript presents interesting descriptive data on drug-resistant TB among patients with T2DM. However, greater critical distance in the interpretation of results, clearer acknowledgment of selection and analytical limitations, and improved internal coherence would substantially strengthen the manuscript. Emphasizing the exploratory nature of the associations and avoiding over-interpretation would better align the conclusions with the data presented.

Reviewer #2: After the revision of the manuscript titled: High Prevalence of Resistance to First-Line Drugs and Multidrug Resistance in

Patients with Tuberculosis and Type 2 Diabetes Mellitus at a Referral Hospital in Peru. Here, the authors aim to to estimate the prevalence of resistance to first-line antituberculosis drugs and identify factors associated with multidrug resistance (MDR) among patients with T2DM and pulmonary TB treated at a public hospital in Lima, Peru. Despite their effort for a statistical analysis there are some points to be improved:

- it is not clear whether there is a diference between the drug resistant TB ( all types) to the drug suceptible gropup.

- Also, author should expand on the rationale on how DM is related to MDR-TB. Both the Introduction and the Discussion, warrant this connecting point.

There is as well other correcions including:

Introduction

• Line 77. Sentence is referring to the incidence of two diseases, but not clear which one.

Methodology

• Line 90. What is the exact population covered by this hospital? this is an important demgraphic data. It seems that this hospital recruits specifically complex cases. If yes, please underline how this setting can however be presented as paradigmatic of the entire MDR-TB problem in Peru.

Analysis plan:

• Please re-write the analysis plan using one whole paragraph. Please make sure to compile all methods correctly.

• Line 155 . Please clarify which method did you use for determining sample adequacy.

Result section:

• Results tables should be at the end of the manuscript.

• The entire section require restructuration and re-writte into adequate paragraph. Its current form only shows spare sentences stating the results. However, there is no connection of ideas among them.

Discussion section

• Line 294 Mycobacterium tuberculosis should always be in italics.

• Lines 302-313 should be a whole entire paragraph.

• Lines 315-325 again should be re-rewirtten into a whole paragraph.

• Lines 327-335 same

• The Discussion section does not provide any putative discussion/explanation/Link between  DM and MDR-TB (why would DM favor MDR-TB rather than Susceptbible TB ?) except that the studied setting. Please insist on this setting-linked limitation.

**********

what does this mean?). If published, this will include your full peer review and any attached files.

If you choose “no”, your identity will remain anonymous but your review may still be made public.

**Do you want your identity to be public for this peer review?**  For information about this choice, including consent withdrawal, please see our Privacy Policy

Reviewer #1: No

Reviewer #2: No

**********

[NOTE: If reviewer comments were submitted as an attachment file, they will be attached to this email and accessible via the submission site. Please log into your account, locate the manuscript record, and check for the action link "View Attachments". If this link does not appear, there are no attachment files.]

To ensure your figures meet our technical requirements, please review our figure guidelines: https://journals.plos.org/plosone/s/figures

You may also use PLOS’s free figure tool, NAAS, to help you prepare publication quality figures: https://journals.plos.org/plosone/s/figures#loc-tools-for-figure-preparation.

NAAS will assess whether your figures meet our technical requirements by comparing each figure against our figure specifications.

# Author comment: Author response to Decision Letter 1

**DOI:** 10.1371/journal.pone.0337152.r002

28 May 2026

PONE-D-25-59435

High Prevalence of Resistance to First-Line Drugs and Multidrug Resistance in Patients with Tuberculosis and Type 2 Diabetes Mellitus at a Referral Hospital in Peru

PLOS One

Dear Editor and Reviewers,

We sincerely thank you for the careful evaluation of our manuscript and for the constructive comments provided. We have revised the manuscript accordingly and believe that these changes have improved the clarity, methodological transparency, and interpretation of the study findings. Below, we provide a detailed point-by-point response to all comments raised

Answer to editor

When submitting your revision, we need you to address these additional requirements.

1. Please ensure that your manuscript meets PLOS ONE's style requirements, including those for file naming. The PLOS ONE style templates can be found at

https://journals.plos.org/plosone/s/file?id=wjVg/PLOSOne_formatting_sample_main_body.pdf and

https://journals.plos.org/plosone/s/file?id=ba62/PLOSOne_formatting_sample_title_authors_affiliations.pdf

Thank you for this observation. We carefully revised the manuscript and supporting files to ensure compliance with PLOS ONE formatting and style requirements

2. Please provide additional details regarding participant consent. In the ethics statement in the Methods and online submission information, please ensure that you have specified (1) whether consent was informed and (2) what type you obtained (for instance, written or verbal, and if verbal, how it was documented and witnessed). If your study included minors, state whether you obtained consent from parents or guardians. If the need for consent was waived by the ethics committee, please include this information.

If you are reporting a retrospective study of medical records or archived samples, please ensure that you have discussed whether all data were fully anonymized before you accessed them and/or whether the IRB or ethics committee waived the requirement for informed consent. If patients provided informed written consent to have data from their medical records used in research, please include this information

Once you have amended this/these statement(s) in the Methods section of the manuscript, please add the same text to the “Ethics Statement” field of the submission form (via “Edit Submission”).

For additional information about PLOS ONE ethical requirements for human subjects research, please refer to http://journals.plos.org/plosone/s/submission-guidelines#loc-human-subjects-research.

Thank you for this observation. We revised the Ethics Statement in the manuscript and submission form to clarify the consent procedures. Because this was a retrospective study based on secondary analysis of anonymized clinical records, the requirement for informed consent was waived by the corresponding ethics committees. No personally identifiable information was accessed during the study.

3. We note that your Data Availability Statement is currently as follows: [All relevant data are within the manuscript and its Supporting Information files.]

Please confirm at this time whether or not your submission contains all raw data required to replicate the results of your study. Authors must share the “minimal data set” for their submission. PLOS defines the minimal data set to consist of the data required to replicate all study findings reported in the article, as well as related metadata and methods (https://journals.plos.org/plosone/s/data-availability#loc-minimal-data-set-definition).

For example, authors should submit the following data:

- The values behind the means, standard deviations and other measures reported;

- The values used to build graphs;

- The points extracted from images for analysis.

Authors do not need to submit their entire data set if only a portion of the data was used in the reported study.

If your submission does not contain these data, please either upload them as Supporting Information files or deposit them to a stable, public repository and provide us with the relevant URLs, DOIs, or accession numbers. For a list of recommended repositories, please see https://journals.plos.org/plosone/s/recommended-repositories.

If there are ethical or legal restrictions on sharing a de-identified data set, please explain them in detail (e.g., data contain potentially sensitive information, data are owned by a third-party organization, etc.) and who has imposed them (e.g., an ethics committee). Please also provide contact information for a data access committee, ethics committee, or other institutional body to which data requests may be sent. If data are owned by a third party, please indicate how others may request data access.

Thank you for this observation. We agree with the importance of data transparency and reproducibility. Accordingly, we prepared and uploaded a de-identified minimal dataset containing the variables required to replicate the analyses reported in the manuscript. The Data Availability Statement was updated to indicate that these data are provided as Supporting Information files.

4. If the reviewer comments include a recommendation to cite specific previously published works, please review and evaluate these publications to determine whether they are relevant and should be cited. There is no requirement to cite these works unless the editor has indicated otherwise.

Not response required

Answer to reviewers

Reviewer 1

5. Introduction: The introduction adequately places the study within the broader global and regional context of tuberculosis.

6. Methods: The study population is drawn from a single, highly specialized referral centre that manages complex TB cases, including drug-resistant TB and patients with comorbidities. While this is clearly stated, the implications of this selection are insufficiently addressed throughout the manuscript. For example, the very high prevalence of MDR-TB and prior TB treatment (>80%) strongly suggests that the study population is not representative of the broader TB-DM population.

Thank you for this important observation. We agree that the study population was drawn from a specialized referral center for complex and drug-resistant tuberculosis cases, which may have concentrated patients with previous treatment exposure and higher baseline probabilities of drug resistance. To address this limitation, we clarified throughout the manuscript that the prevalence estimates reported should not be interpreted as representative of the overall TB-DM population in Peru, and we emphasized the limited generalizability of the findings in the Discussion section.

7. Regarding the four main anti-TB drugs, the fourth is pyrazinamide, not streptomycin, please clarify.

Thank you for this observation. We agree that pyrazinamide, rather than streptomycin, is considered one of the four main first-line anti-tuberculosis drugs. The error was limited to the Methods section describing drug susceptibility testing, while the classification and reporting in the tables were already correct. The corresponding section has been revised for consistency.

8. Diabetes treatment regimen classification is different between Methods section and Results section, please clarify.

Thank you for this observation. We standardized the diabetes treatment categories throughout the manuscript as: diet only, metformin only, basal insulin ± metformin, and basal–bolus insulin regimen.

Results:

9. Line 360: The authors mention “360 patients diagnosed with both diabetes and tuberculosis” during the study period. However, this number of patients is not mentioned anywhere else in the manuscript, not even in Figure 1. Please clarify.

Thank you for this observation. We clarified this information in the Results section and Figure 1. Specifically, 189 corresponds to the total number of unique patients diagnosed with both diabetes and tuberculosis during the study period, before applying the eligibility criteria. After exclusions, 130 patients with complete susceptibility data for MDR-TB classification were included in the analytical sample.

10. Since the data in the "Total sample (N=144)" column of Table 1 are not mentioned in the text nor used anywhere in the manuscript, please delete this column or clarify how this data is relevant.

Thank you for this observation. The “Total sample (N=144)” column was initially included for transparency purposes, as only 130 patients had complete susceptibility data required for MDR-TB classification. To improve clarity and focus on the analytical sample used in the main analyses, we moved the comparison table to the Supplementary Material and retained only the final analytical sample (N=130) in the main manuscript.

11. In Table 2, authors used HRZE abbreviations for anti-TB drugs but did not define all of them in the Table footnote. Furthermore, the abbreviation Z refers to pyrazinamide, but according to the Methods section, susceptibility to this antibiotic was not evaluated. Please clarify.

Thank you for this observation. We clarified all anti-tuberculosis drug abbreviations used in Table 2 and its footnotes, including isoniazid (H), rifampicin (R), pyrazinamide (Z), and ethambutol (E). We also corrected the Methods section to clarify that pyrazinamide susceptibility, rather than streptomycin susceptibility, was evaluated.

12. The authors analyse two outcomes: “Any drug resistance” and “MDR-TB”. However, since the MDR-TB category is included within the category any drug resistance, these two outcomes should not be treated and discussed separately in the main text.

Thank you for this observation. We clarified throughout the manuscript that MDR-TB represents a subgroup within the broader category of resistance to at least one first-line drug. The Results and Discussion sections were revised to avoid interpreting these outcomes as independent or mutually exclusive categories.

13. In Table 3, percentages appear to be calculated by row rather than by column. While this presentation is mathematically correct, it makes it difficult to visually assess differences in the distributions between drug resistant positive and negative groups. A column-wise presentation (showing for example the distribution of age groups within each MDR-TB category) would be more intuitive and better aligned with the interpretation of Pearson’s chi-square test.

Thank you for this observation. We agree that column-wise presentation facilitates interpretation of the distribution of characteristics according to resistance status and is more consistent with the interpretation of Pearson’s chi-square test. Therefore, Table 3 was revised to present percentages by column instead of by row.

14. Lines 218-224: The authors say the same thing twice, just in different ways. Moreover, the youngest age group is not 40-49 years. Please rephrase. Please clarify the choice of variables for multivariable analysis.

Thank you for this observation. We revised this section to remove redundant statements and clarify the interpretation of the age groups. We also clarified the variable selection strategy for the multivariable models. Given the limited number of MDR-TB cases and the exploratory nature of the study, the adjusted models included a restricted number of variables selected according to clinical relevance and strength of association in the bivariable analysis to reduce the risk of overfitting.

Discussion:

15. The limitation related to recruitment from a specialized referral centre should be considered throughout the entire Discussion, rather than as an isolated point. This setting inherently selects for complex and drug-resistant TB cases, introducing substantial referral bias that affects prevalence estimates, observed associations, and their interpretation. The authors must revisit the entire discussion, systematically taking into account that this bias risk leading to an overinterpretation of the results and an extrapolation of the conclusions beyond the studied population. Moreover, in several places, the Discussion moves toward mechanistic explanations (immunosenescence, cumulative exposure, metabolic alterations) without sufficiently emphasizing the exploratory and observational nature of the findings.

Thank you for this important observation. We agree that the specialized referral setting may have introduced referral bias by concentrating patients with more complex and drug-resistant TB presentations. Accordingly, we revised the Discussion section throughout the manuscript to more consistently acknowledge that the prevalence estimates and observed associations may not be representative of the overall TB-DM population and should be interpreted with caution.

We also revised several sections of the Discussion to reduce overinterpretation of the findings and better emphasize the exploratory and observational nature of the study. In particular, potential biological mechanisms were reframed as possible hypotheses rather than causal explanations.

16. Lines 255-256: The sentence “This study documents a high frequency of bacterial resistance to first-line anti-tuberculosis drugs among patients with tuberculosis and type 2 diabetes mellitus (TB-DM) co-infection” implies a comparative interpretation that is not directly supported by the study design. In the absence of a TB-only control group, the study cannot determine whether resistance is higher in TB–DM patients than in TB patients without diabetes. Moreover, recruitment from a specialized referral centre managing complex and drug-resistant TB cases introduces substantial selection bias, limiting the generalizability of the reported resistance frequency. Please rephrase.

Thank you for this observation. We agree that the original wording could be interpreted as implying a comparison with patients without diabetes, which is not supported by our study design. Therefore, we revised the Discussion section to better reflect the descriptive and exploratory nature of the study. We also clarified throughout the Discussion that the study was conducted at a specialized referral center for complex and drug-resistant tuberculosis cases, which may limit the generalizability of the findings to the overall TB-DM population.

17. Lines 270-271: Author state that: “However, the populations, levels of care, and methodologies included in those studies could explain the differences with our research.” Please elaborate further on this key point for the analysis of the study in light of the literature.

Thank you for this observation. We agree that the original statement was too general. We revised the Discussion section to further explain that the studies differed in patient characteristics, healthcare settings, and methodological approaches. In particular, our study was conducted at a specialized referral center for complex and drug-resistant tuberculosis cases, which likely concentrated patients with previous treatment exposure and higher baseline probabilities of drug resistance.

18. Lines 278-279: The manuscript refers to individuals aged 45-64 years as “young adults.” This terminology is misleading, as this age range is generally classified as middle-aged rather than young adulthood in epidemiological and clinical literature. Please use more appropriate age descriptors.

Thank you for this observation. We agree that the term “young adults” was not appropriate for the 45–64-year age group. Accordingly, we revised the manuscript to use the more appropriate term “middle-aged adults.”

19. Line 346: The wording “clarified resistance patterns” is misleading. Multivariable analysis in this study does not resolve underlying resistance patterns but rather identifies exploratory associations within a selected referral population. Please rephrase.

Thank you for this observation. We agree that the original wording could imply definitive interpretation of resistance patterns. Therefore, we revised the sentence to better reflect the exploratory nature of the multivariable analysis and the characteristics of the referral population. The sentence was rephrased to indicate that the adjusted analysis identified factors associated with MDR-TB within this specialized referral population.

20. Lines 358-361: Please specify more cle

Attachment Submitted filename: Answer to reviewers - MDR.docx

# Peer review: Decision Letter 1

**DOI:** 10.1371/journal.pone.0337152.r003

**Authors:** Frederick Quinn

7 Aug 2026

High Prevalence of Resistance to First-Line Drugs and Multidrug Resistance in Patients with Tuberculosis and Type 2 Diabetes Mellitus at a Referral Hospital in Peru

PONE-D-25-59435R1

Dear Dr. Yovera-Aldana ,

We’re pleased to inform you that your manuscript has been judged scientifically suitable for publication and will be formally accepted for publication once it meets all outstanding technical requirements.

Within one week, you’ll receive an e-mail detailing the required amendments. When these have been addressed, you’ll receive a formal acceptance letter and your manuscript will be scheduled for publication.

An invoice will be generated when your article is formally accepted. Please note, if your institution has a publishing partnership with PLOS and your article meets the relevant criteria, all or part of your publication costs will be covered. Please make sure your user information is up-to-date by logging into Editorial Manager at Editorial Manager® and clicking the ‘Update My Information' link at the top of the page. For questions related to billing, please contact billing support.

If your institution or institutions have a press office, please notify them about your upcoming paper to help maximize its impact. If they’ll be preparing press materials, please inform our press team as soon as possible -- no later than 48 hours after receiving the formal acceptance. Your manuscript will remain under strict press embargo until 2 pm Eastern Time on the date of publication. For more information, please contact onepress@plos.org.

Kind regards,

Frederick Quinn

Academic Editor

PLOS One

Additional Editor Comments (optional):

Reviewers' comments:

Reviewer's Responses to Questions

**Comments to the Author**

Reviewer #1: All comments have been addressed

**********

2. Is the manuscript technically sound, and do the data support the conclusions?

Reviewer #1: Yes

**********

3. Has the statistical analysis been performed appropriately and rigorously?

Reviewer #1: Yes

**********

4. Have the authors made all data underlying the findings in their manuscript fully available?

The PLOS Data policy

Reviewer #1: (No Response)

**********

5. Is the manuscript presented in an intelligible fashion and written in standard English?

Reviewer #1: Yes

**********

Reviewer #1: The authors have generally addressed the main points raised during the first round of review, and the revisions improve the clarity and consistency of the manuscript. A few remaining typographical and minor formatting errors are still present, but these can be resolved during the editorial processing stage.

**********

what does this mean?). If published, this will include your full peer review and any attached files.

If you choose “no”, your identity will remain anonymous but your review may still be made public.

**Do you want your identity to be public for this peer review?**  For information about this choice, including consent withdrawal, please see our Privacy Policy

Reviewer #1: No

**********

# Editor report: Acceptance letter

**DOI:** 10.1371/journal.pone.0337152.r004

**Authors:** Frederick Quinn

PONE-D-25-59435R1

PLOS One

Dear Dr. Yovera-Aldana,

I'm pleased to inform you that your manuscript has been deemed suitable for publication in PLOS One. Congratulations! Your manuscript is now being handed over to our production team.

At this stage, our production department will prepare your paper for publication. This includes ensuring the following:

* All references, tables, and figures are properly cited

* All relevant supporting information is included in the manuscript submission,

* There are no issues that prevent the paper from being properly typeset

You will receive further instructions from the production team, including instructions on how to review your proof when it is ready. Please keep in mind that we are working through a large volume of accepted articles, so please give us a few days to review your paper and let you know the next and final steps.

Lastly, if your institution or institutions have a press office, please let them know about your upcoming paper now to help maximize its impact. If they'll be preparing press materials, please inform our press team within the next 48 hours. Your manuscript will remain under strict press embargo until 2 pm Eastern Time on the date of publication. For more information, please contact onepress@plos.org.

You will receive an invoice from PLOS for your publication fee after your manuscript has reached the completed accept phase. If you receive an email requesting payment before acceptance or for any other service, this may be a phishing scheme. Learn how to identify phishing emails and protect your accounts at https://explore.plos.org/phishing.

If we can help with anything else, please email us at customercare@plos.org.

Thank you for submitting your work to PLOS One and supporting open access.

Kind regards,

PLOS ONE Editorial Office Staff

on behalf of

Dr. Frederick Quinn

Academic Editor

PLOS One
